# An atypical and bilateral presentation of Charcot foot disease

**DOI:** 10.1186/s12902-019-0422-z

**Published:** 2019-09-05

**Authors:** C. V. Loupa, E. Meimeti, A. Kokas, E. D. Voyatzoglou, A. Donou

**Affiliations:** 1 Demetrios Voyatzoglou Diabetic Foot Clinic, Amalia Fleming Hospital Unit, 14, 25th of March st., Melissia, GR-15127 Athens, Greece; 2 Radiology Department, Amalia Fleming Hospital Unit, Athens, Greece

**Keywords:** Charcot foot disease, Neuropathic osteoarthropathy, Bilateral, Diabetes mellitus, Diabetic foot infection, Ankle oedema, Foot deformity

## Abstract

**Background:**

Charcot neuropathic osteoarthropathy (CNO) is one of the most devastating complications of neuropathy in patients with diabetes. Establishing diagnosis of CNO is difficult, due to the lack of clear clinical and radiological diagnostic criteria. Diagnosis is even more difficult when there is atypical and bilateral clinical presentation. Since CNO may lead to foot deformity, lower-extremity amputation and significant decrements in quality of life, it must be detected and treated without delay. Treatment focuses mainly on interruption of the inflammatory process and relief from pain using feet offloading devices. In more severe cases, surgical intervention may be needed. Additionally, the use of custom-made insoles and custom-made orthopaedic shoes is mandatory.

**Case presentation:**

We report a case of a young diabetic patient who presented to our clinic with bilateral and atypical presentation of Charcot foot disease. Patient was treated successfully upon diagnosis with bilateral aircast offloading. Unfortunately, due to depression and non-compliance, the disease progressed to severe and permanent lesions later on.

**Conclusion:**

Despite the rareness of this disease, clinicians must include CNO into differential diagnosis of diabetic foot oedema, inflammation and deformity.

## Background

Charcot neuropathic osteoarthropathy (CNO) is one of the most devastating complications of neuropathy. In the Western world, CNO mainly occurs in the feet of patients with diabetes. The prevalence of diabetic CNO of the foot is difficult to determine due to the lack of clear clinical and radiological diagnostic criteria [1, 2].

Pathogenesis of CNO includes local, but not systemic, inflammation [3], that is triggered by a minor injury, infection, surgery, or an earlier ulceration [4], increased osteopenia, and increased expression of the polypeptide receptor activator of nuclear factor-kappaB ligand (RANKL) [5]. Neuropathy is an universal and essential feature, while patients have well preserved limb arterial blood flow, at least at the early stages [1].

Because CNO is relatively rare and most patients present unilateral symptoms with significant bone pathology, redness, swelling, warmth and pain, CNO is often misdiagnosed as cellulitis, deep venous thrombosis, trauma or gout and remain untreatable. Also, many cases of CNO are misdiagnosed as diabetic foot osteomyelitis [6], and this results to useless long-time antibiotic treatment and time loss, causing irreversible foot lesions. Unfortunately, in the first months of the disease, radiological findings are absent or subtle. These first months are crucial for therapy, because if Charcot foot is treated early with proper offloading, the disease will be controlled and there will be no changes or deformities. Thus, CNO must be detected and treated rapidly [7]. Otherwise, specific surgical operations like external or internal fixation and osteotomy may be required, and quality of life may be deteriorated [1].

Moreover, the early diagnosis of CNO may be difficult in patients who present bilateral symptoms of CNO such as foot swelling, erythema and elevated foot temperature.

The treatment steps of CNO is to stop the inflammatory process, relieve pain and minimise potential foot deformity with the use of mechanical protection. Total contact casting of the affected limb consists the gold standard of therapy, although other pressure offloading and immobilisation devices as aircast can be used [8]. Surgical intervention is often required at the later stages.

We present a case of CNO in a young diabetic patient which was at first misdiagnosed due to its bilateral and atypical clinical presentation.

## Case presentation

A 28-year-old woman, with a history of type 1 diabetes mellitus (HbA1c = 14,9%) since the age of 12, poorly controlled despite intensified insulin treatment, who worked as a salesperson in a ladies garment department, which entailed long standing hours, was referred to our Diabetic Foot Clinic due to severe symmetrical oedema in both feet/ankles for at least 8 months without fever or other joint swelling. There was no history of recent trauma. Her body mass index (BMI) was 22,5 kg/m2, and she was non-smoker. She was not taking any other medicine except insulin. She had nothing to mention in her past medical history except two episodes of complicated urinary tract infections.

The patient had been hospitalised few months ago in another hospital for the same reason without been diagnosed.

**Fig. 1:**
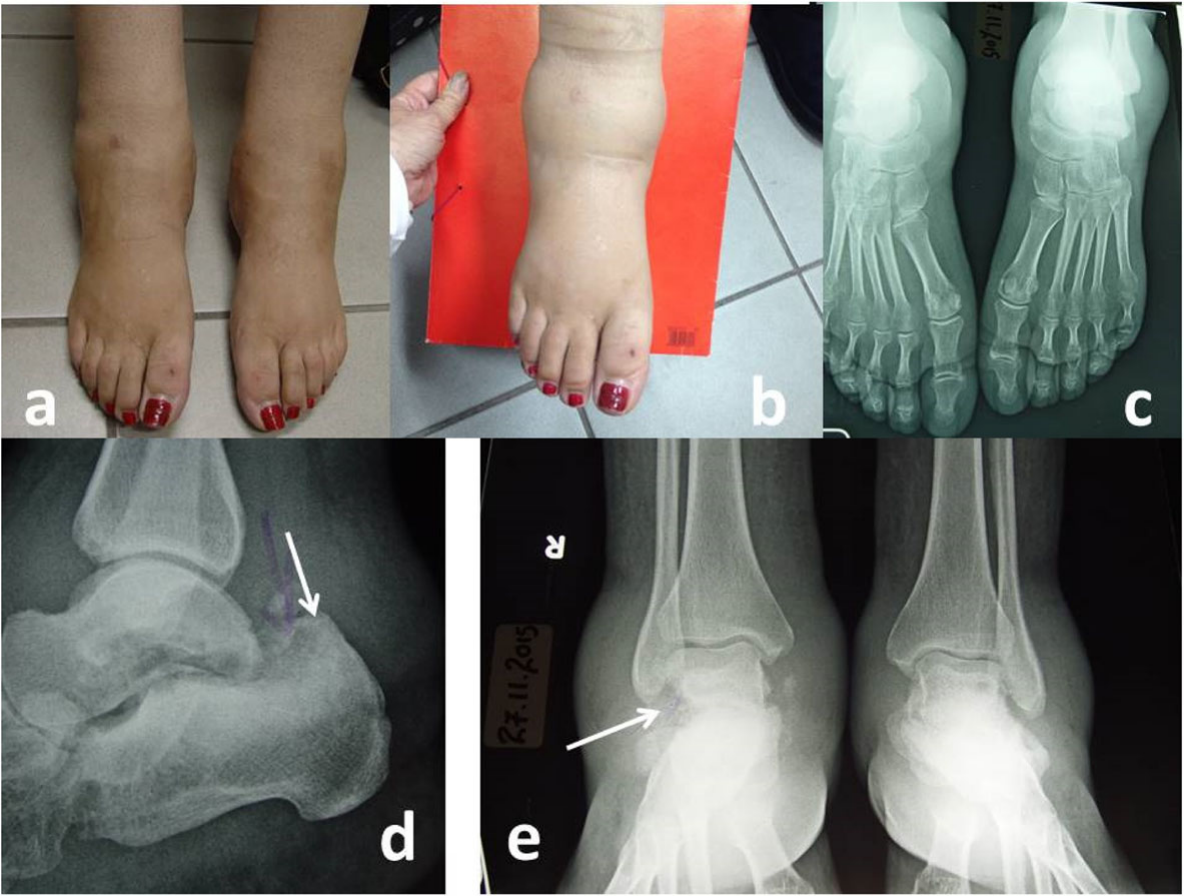
**a**, **b** Extensive oedema of both ankles. **c**-**e** x-rays: Pathological findings of Charcot in both feet, more severe on the right, involving erosion of the upper surface of the calcaneus and the outer surface of the talus (arrows). Also free floating bone segments in the adjoining soft tissues. Bilateral soft tissue oedema

Upon clinical examination, she had excessive bilateral ankle swelling (Fig. 1a, b). Both ankle joints were warm, but she had no foot ulceration, open wounds, or other deformity. Both feet had a loss of sensation, with abnormal monofilament and biothesiometer examination, as well as abnormal Neuropad® test. Thermal sensitivity was not evaluated. The peripheral arterial foot pulses were normal.

Except from the abnormal Neuropad test, she did not have any other symptoms or signs of autonomic neuropathy.

Blood tests showed normal white blood cells (WBC) count (6900/μL) and slightly elevated erythrocyte sedimentation rate (ESR): 56 mm/h, and C-reactive protein (CRP): 16,4 mg/L (normal values < 3). The levels of serum albumin, serum creatinine, transaminases, uric acid and electrolytes were between normal ranges. Urine microalbumin test did not detect albuminuria. Abdominal ultrasound and echocardiogram were normal. Funduscopy revealed background retinopathy.

Main physical and laboratory characteristics of the patient are summarized in Table 1.

**Table 1:** Main clinical and laboratory parameters at the different visits

Parameter	Visit 1	Visit 2	Visit 3	Visit 4	Lost-to-follow-up (23 months)	Visit 5
Time lapse from Visit 1	0	1 month	3 months	5 months		32 months
Demographics: woman	28 yrs. old					31 yrs. old
BMI (kg/m^2^)	22,5	22,5	22,5	22,5		23,0
DM treatment	Insulin degludec / Insulin aspart	same	same	same		Towards CSII
HbA1c	14,9%	NA^a^	9,8%	11%		8%
Creatinine / urea^b^	0,69 / 34	NA^a^		0,7 / 39		1,0 / 47 But recent nephrotic syndrome
WBC (/μL)	6900	NA^a^	7210	7700		8220
ESR (mm/1 h)	56	NA^a^	42	26		40
CRP^c^	16,4	NA^a^	11,2	4,37		13,2
Retinopathy	+	+	+	+		+++
Neuropathy, peripheral^d^	+	+	+	+		+
Neuropathy, autonomic	+	++	++	++		++
Ankle oedema	+++	+	+	++		++
X-ray findings	+		+	++		+++

^a^NA = non applicable

^b^Normal values: creatinine < 1,2 mg/dL, urea< 50 mg/dL

^c^Normal values: < 3 mg/L

^d^Based on clinical examination: monofilament, biothesiometer, Neuropad®

Based on the above biochemical and urimalysis results, nephrotic syndrome had to be ruled out.

In the color Doppler sonography (CDS), the only pathological finding was highly elevated blood flows in the proximal tibial and dorsal arteries, which were attributed to increased pressure on the above mentioned vessels to the excessive oedema and to inflammation.

Plain X-rays revealed bilateral soft tissue oedema, heel osteolyses, and free bone segments, findings compatible with Charcot disease in both feet (Fig. 1c-e). Ordering MRI was not considered necessary, since X-ray findings were revealing.

**Fig. 2:**
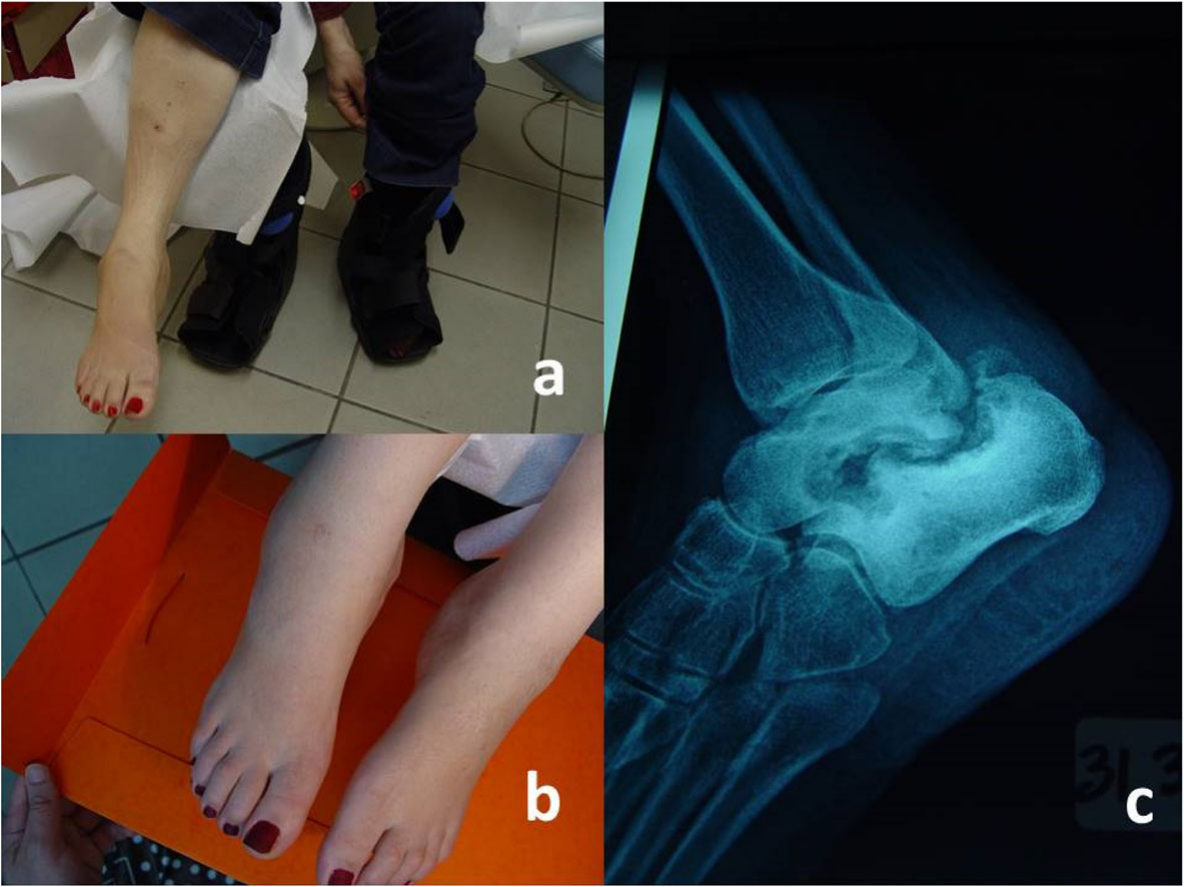
**a** Clinical improvement after 3 months of offloading using aircast devices on both feet. **b**, **c** relapse of the oedema after another 2 months, and worsening of the existing radiological findings with osteoporosis because of weightlessness

Treatment plan included immediate offloading of both feet by the use of an aircast type device. She was advised to rest and stop working standing, if possible. Aircast was put on (Fig. 2a) and she was clinically evaluated four weeks after initiation of treatment and monthly afterwards, in order to ensure no recurrence of the disease. Patient had not painful meuropathy, so pharmacological treatment was not considered.

During follow-up period, oedema seemed to retreat and radiological lesions remained stable. After 3 months of conservative treatment, the patient presented a satisfactory clinical improvement (Fig. 2a). Additionally, no episodes of ulceration were mentioned.

Unfortunately, after another 2 months, oedema seemed to worsen, probably because of non-compliance (Fig. 2b). She was wearing aircasts intermittently, and she was walking long distances using tennis shoes. Patient had depression, her HbA1c was 11% and presented tachycardia, probably attributable to autonomic neuropathy. X-ray findings had worsened (Fig. 2c). Medical situation was explained to her in detail. She was asked to wear aircast devices continuously, and at least to wear custom-made shoes when not in aircasts, in order to distribute weight pressure equally across sole. Also, psychological support was demanded. Patient remained non-complient for the next 2 months. We considered irremovable total contact cast, but then she was lost to follow-up.

**Fig. 3:**
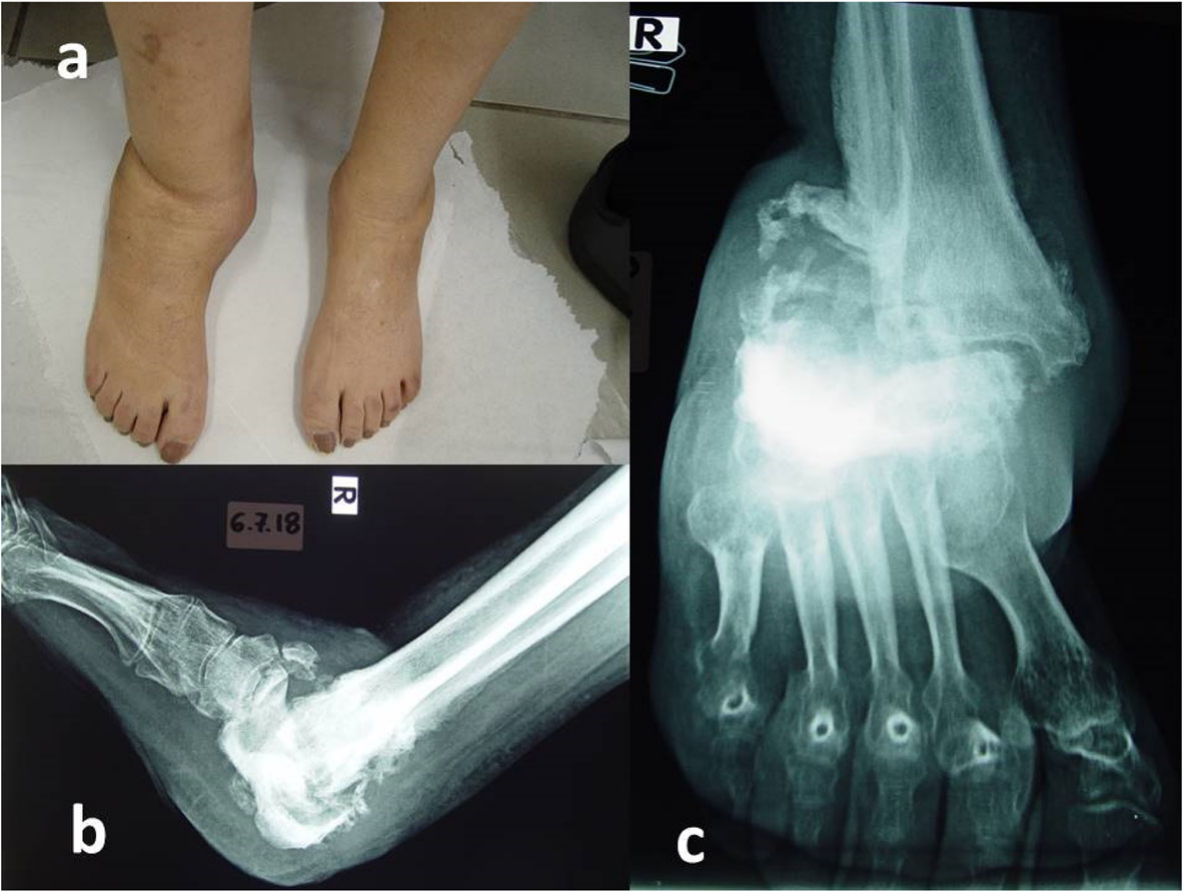
Two years later. **a** Excessive oedema and dislocation of right ankle. **b**, **c** X-rays: further bony destruction of the calcaneus and talus. Severe subluxation of tibial-tarsal joint. Presence of air striations of foot and lower third of extremity, possibly indicating non-aerobial infection. Further osteoporosis of the metatarsal bones

She revisited our foot clinic 2 years later. She was then 31 years old, on depression medications, and her HbA1c was 8%. She reported intraocular haemorrage, foot infections and nephrotic syndrome, due to which she was hospitalized several times. She had severe deformities of both feet. Lesions were more prominent in the right ankle and foot, with joint being dislocated and warm (Fig. 3a). She had further bony destruction of calcaneus and talus upon X-rays (Fig. 3b, c). She was advised to visit a specialized Charcot surgery center, since no conservative treatment was considered to be helpful.

## Discussion and conclusion

First described by JM Charcot in 1886 [9], it is now considered an inflammatory syndrome and is one of the most devastating complications of neuropathy in patients with diabetes in the Western world [1, 2].

It is difficult to diagnose CNO due to its rareness and its symptoms that resemble cellulitis, deep venous thrombosis, trauma or gout [7]. Of course there are the atypical forms of CNO, that can worsen the problem.

On the other hand, the presence of CNO is a serious and limb-threatening lower-extremity complication of diabetes, that can lead to chronic foot deformity or limb amputation. Charcot foot lesions resemble to those after a 9-storey-fall [10], as expert on the field Nina Petrova says, and finally foot looks like a bag of bones in the plain X-ray, as another expert, Andrew JM Boulton, first stated.

CNO treatment hallmark is trying to stop inflammatory process and relieve pain by offloading feet. Custom-made insoles and custom-made orthopaedic shoes may follow [5]. Some times, surgery may be the only treatment available.

A lot of published studies have been demonstrate the pathophysiology, diagnosis algorithm and treatment management of CNO, but only few studies report bilateral symptoms or atypical presentations of feet/ankle CNO [11–14]. It has to be noted that bilateral CNO is reported in only 12% of Charcot patients [14].

This case report demonstrates an impressive bilateral and atypical presentation of Charcot foot disease in a young woman. Radiological findings of both feet were evident at presentation, since CNO existed for at least 8 months before first visit to our clinic. Peripheral neuropathy, essential for CNO pathogenesis, was present (monofilament, biothesiometer and Neuropad® test), and limb arterial flow was normal, with elevated blood flows. Patient was given the option of offloading one foot first, but she decided to put aircast devices in both feet. Oedema subsided after the first months of offloading, but then she presented with depression because of devastated quality of life, and she was non-complient. When she reappeared after 2 years of lost-to-follow-up, clinical and radiological changes were so prominent that only surgical treatment was possible, and this with doubtful results.

Therefore, we believe that it is worth reporting the above CNO case, in order to awake medical society about this disease and include CNO into differential diagnosis of foot oedema in diabetic patients, despite the rareness of the disease. Also, it has to be underlined how important is psychological support, especially when CNO appears in young diabetic patients.

In conclusion, we report a case of CNO where atypical and bilateral symptoms of foot and ankle were present. The long time that had elapsed until the diagnosis allowed radiological findings to be present in plain x-rays at patients’ first visit to our clinic; so MRI was not considered necessary. Unfortunately, due to non-compliance, progression of the disease resulted to permanent and prominent lesions.

## Data Availability

The datasets used and/or analysed during the current study are available from the corresponding author on reasonable request.

## Abbreviations

BMI: Body mass index
CDS: Color doppler sonography
CNO: Charcot neuropathic osteoarthropathy
CRP: C-reactive protein
ESR: Erythrocyte sedimentation rate
WBC: White blood cells
